# Determining the metabolic impact of postbiotics in mice

**DOI:** 10.1016/j.xpro.2021.101098

**Published:** 2022-01-13

**Authors:** Fernando F. Anhê, Arshpreet Bhatwa, Jonathan D. Schertzer

**Affiliations:** 1 Department of Biochemistry and Biomedical Sciences, McMaster University, 1200 Main St. W., Hamilton, Ontario L8N 3Z5, Canada; 2 Farncombe Family Digestive Health Research Institute, McMaster University, 1200 Main St. W., Hamilton, Ontario L8N 3Z5, Canada; 3 Centre for Metabolism, Obesity and Diabetes Research, McMaster University, 1200 Main St. W., Hamilton, Ontario L8N 3Z5, Canada

**Keywords:** Health Sciences, Immunology, Metabolism, Microbiology, Model Organisms

## Abstract

Postbiotics cooperate to influence immune and metabolic outcomes in the host. Here we describe a protocol for *in vivo* assessment of blood glucose control following acute administration of lipopolysaccharide (LPS) and peptidoglycan (PGN) in mice. This protocol can be adapted for testing a broad range of microbial molecules and ligands for host immune receptors. Experience with mouse handling is required.

For complete details on the use and execution of this protocol, please refer to Anhê et al. (2021) and Cavallari et al. (2017).

## Highlights

• Postbiotics influence host immunometabolism and change blood glucose control
• Our model tests synergy or tolerance of postbiotics on blood glucose *in vivo*
• Lipopolysaccharide (LPS) and peptidoglycan (PGN) are used as examples
• A glucose tolerance test (GTT) is one suitable endpoint

## Graphical abstract

**Figure:**
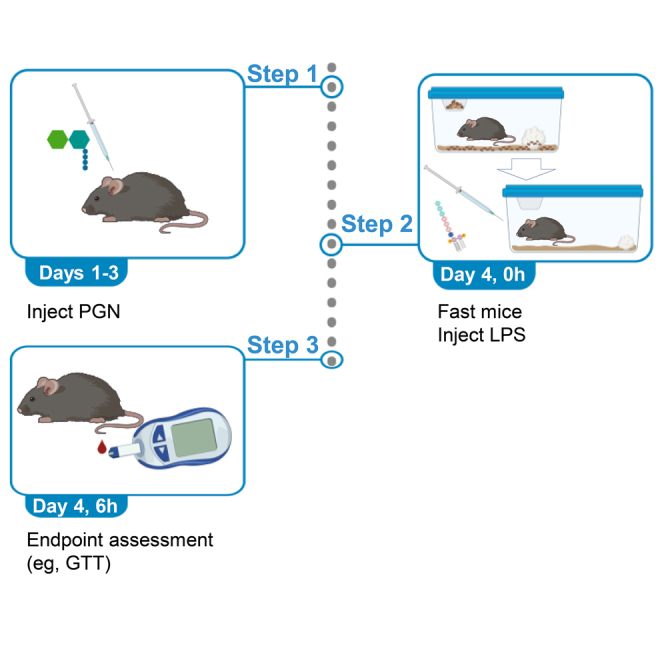

## Before you begin

**Timing: 1–2 weeks**

We present a protocol for testing the impact of postbiotics on host metabolism *in vivo*, where examples are peptidoglycan (PGN) working in synergy or promoting tolerance when combined with lipopolysaccharide (LPS) to alter blood glucose control. Our exemplar approach used meso-diaminopimelic acid (DAP)-PGN or muramyl dipeptide (MDP)-PGN and hexa-acylated LPS from *Escherichia coli*. This protocol allows testing the metabolic impact of postbiotics using a simple and efficient technique. This method also models the synergy or tolerance that can occur between postbiotics, which can provide insight into the interaction of microbial-derived molecules on host metabolic regulation. This protocol can be easily adapted to different types of PGN and/or LPS in addition to other postbiotics. However, adaptations using other postbiotics may require optimization, especially regarding the dose and timing of administration. Likewise, while this protocol describes the assessment of blood glucose control by means of a glucose tolerance test (GTT) following PGN and LPS administration, the main metabolic endpoint may also be adapted. Metabolic tests other than a GTT (i.e., insulin, pyruvate or lipid tolerance test) may require fasting strategies different from the one described herein.

Animals: Ensure that all animal procedures were institutionally approved and comply with local ethics board guidelines. Mice procured from a vendor should be acclimatized for at least 1–2 weeks prior to testing. Different treatment groups must be matched for weight and age. Sex, strain, diet, room temperature, housing room, and number of mice per cage must be consistent across treatment groups and cohorts. Mice must be in a 12h light/dark cycle and have access to food and water *ad libitum*. The present protocol was optimized for 12–25-week-old C57Bl6N or C57Bl6J mice. Testing of ten to twelve mice per group is recommended as the minimum sample size.

Access to a software that allows graphical visualization of data and statistical analysis is necessary. We recommend GraphPad Prism version 8 or 9.

Postbiotics: Whenever possible, we recommend the use of ultrapure grade LPS. Several LPS isolates that are commercially available have considerable amounts of other Toll-like receptor (TLR) ligands, which can be an important confounding factor (Parusel et al., 2017). Ensure to use endotoxin-free water to prepare LPS and PGN stock aliquots at 1 mg/mL, keep them at -20°C and choose a volume that minimizes repeated freeze-thaw cycles. We recommend 0.5–1.0 mL aliquots. To inject LPS or the NOD1 agonist FK565 to 30 mice weighing ∼30 g, ∼0.5 mL of stock solution is needed; for MDP, ∼3.0 mL is needed. Adjust the volume of aliquots taking into account the approximate volume needed for each experiment. Prepare aliquots in MAXYMum Recovery® tubes (or similar) and always vortex them vigorously for at least 15 min before use to mitigate binding of ligands to plastic. We recommend the use of a ThermoMixer C® or similar benchtop shaker to allow consistent vortexing.

Blood glucose assessment: To avoid discrepancies in absolute blood glucose readings, ensure to use glucometers and glucose strips from the same brand/model across different experiments.

## Key resources table

**Table:** 

REAGENT or RESOURCE	SOURCE	IDENTIFIER
**Chemicals, peptides, and recombinant proteins**		

Ultrapure LPS *Escherichia coli* O111:B4	InvivoGen	Cat# tlrl-3pelps
Ultrapure LPS *Rhodobacter sphaeroides*	InvivoGen	Cat# tlrl-prslps
Ultrapure LPS *Salmonella minnesota* R595	InvivoGen	Cat# tlrl-smlps
Ultrapure LPS *Porphyromonas gingivalis*	InvivoGen	Cat# tlrl-ppglps
Peptidoglycan MDP	InvivoGen	Cat# tlrl-mdp
Peptidoglycan FK565	Astellas Pharma	Not available
Peptidoglycan iE-DAP	InvivoGen	Cat# tlrl-dap
Peptidoglycan C14-Tri-LAN-Gly	InvivoGen	Cat# tlrl-ctlg
Teklad 22/5 Rodent Diet (chow, 17% kcal from fat)	Envigo	Cat# 8640
Glucometer and glucose monitoring strips	MediSure	Not available

**Experimental models: Organisms/strains**		

Mouse C57BL/6NTac (male, 12-25-week-old)	Taconic	B6-M
Mouse C57BL/6J (male, 12-25-week-old)	The Jackson Laboratory	Cat# 000664

**Software and algorithms**		

GraphPad Prism 8 or 9	GraphPad	https://www.graphpad.com/

## Materials and equipment

**Table:** NOD1 agonist PGN FK565 solution

Reagent	Final concentration	Amount
FK565 (1 mg/mL)	**50 μg/mL**	10 μL
Sterile saline	n/a	190 μL
**Total**	**n/a**	**200 μL**

***Note:*** Do not store this solution. A fresh solution must be prepared before injection. 200 μL of a 50 μg/mL solution is the volume necessary to inject 1 mouse with a dose of 10 μg/mouse∗.

**Table:** NOD2 agonist PGN MDP solution

Reagent	Final concentration	Amount
MDP (1 mg/mL)	**500 μg/mL**	100 μL
Sterile saline	n/a	100 μL
**Total**	**n/a**	**200 μL**

***Note:*** Do not store this solution. A fresh solution must be prepared before injection. 200 μL of a 500 μg/mL solution is the volume necessary to inject 1 mouse with a dose of 100 μg/mouse∗.

**Table:** LPS solution

Reagent	Final concentration	Amount
*E. coli* LPS (1 mg/mL)	**0.04 mg/mL**	10 μL
Sterile saline	n/a	240 μL
**Total**	**n/a**	**250 μL**

***Note:*** Do not store this solution. A fresh solution must be prepared before injection. If testing the synergy between LPS and a NOD1 ligand, use 250 μL of a LPS solution at 0.04 mg/mL to inject 1 mouse weighing 25 g (this yields a dose equivalent to 0.4 g/kg). However, if testing LPS in synergy with a NOD2 ligand, use 125 μL of a LPS solution at 0.04 mg/mL to inject 1 mouse weighing 25 g (this equates to a 0.2 g/kg dose)∗.

**Table:** D-glucose solution

Reagent	Final concentration	Amount
D-glucose	**0.2 g/mL**	2 g
Sterile saline	n/a	Top to 10 mL
**Total**	**n/a**	**10 mL**

***Note:*** Do not store this solution. A fresh solution must be prepared before injection. 10 mL of a solution at 0.2 g/mL is sufficient to inject 33 mice weighing 30 g using a 2 g/kg dose∗.

∗Note that the dose of PGN is not corrected for body weight and is calculated per mouse. Conversely, LPS and glucose are corrected for body weight and doses are calculated in g/kg.

When calculating the total volume needed, we recommend preparing ∼25% excess to account for the needle’s dead volume.

***Alternatives:*** FK565 was provided by Astellas Pharma and can be difficult to obtain or synthesize. Other NOD1 agonists (eg, iE-DAP, C14-Tri-LAN-Gly) are commercially available. These NOD1 ligands can serve as an alternative to FK565 for *in vitro* experiments. However, in our experience FK565 (+LPS) is required for profound changes in blood glucose *in vivo*. It is not yet clear why FK565 is such as potent NOD1 ligand to cause dysglycemia *in vivo* compared to other NOD1 ligands. iE-DAP and C14-Tri-LAN-Gly may be less lipophilic than FK565. It is important to consider that NOD1 ligands other than FK565 may also have **different** tissue distribution dynamics, which may limit their dysglycemic potential even at very high doses. Other types of ultrapure grade LPS are also available, such as that of *Rhodobacter sphaeroides*, *Salmonella minnesota* and *Porphyromonas gingivalis*. The same conditions described herein for *E. coli* LPS can be applied to test distinct LPS types.

## Step-by-step method details

### Injection with peptidoglycan

**Timing: 3–4 days**

Repeated injections with PGN are used to engage robust innate immune response.

1. Prepare PGN solution.

Prepare enough volume of a solution containing 50 μg of Nucleotide-binding Oligomerization Domain-containing protein (NOD)1 agonist PGN /mL of sterile saline. For NOD2 agonist PGNs, prepare a solution at 500 μg/mL of sterile saline. Always prepare fresh solutions on the day of injection and do not save remaining volume for the next day.

2. Inject mice with PGN.
  a. Use the intraperitoneal route to deliver 200 μL of NOD1 agonist PGN (10 μg/mouse/day) for 3 consecutive days to half of the cohort. If using NOD2 agonist PGNs, inject 200 μL (i.e., 100 μg/mouse/day) for 3 consecutive days to half of the cohort. The time of the injection must be consistent over the days.
  b. Inject the other half of the cohort with sterile saline.

***Note:*** This protocol was optimized using FK565, a synthetic DAP-containing PGN and potent NOD1 agonist. MDP, the minimal bioactive PGN motif that activates NOD2, was used to optimize the methods for NOD2 agonist PGNs. This protocol is suitable for testing NOD receptor antagonists and postbiotics activating pathways related to innate immune receptors other than NODs. Dose and timing of administration may require some optimization for different postbiotics.

### Injection with lipopolysaccharide

**Timing: 6 h**

This step promotes the synergy between different postbiotics as PGN-treated mice receive one single dose of LPS.

3. Prepare LPS solution.

Prepare enough volume of a solution containing 0.04 mg of *E. coli* LPS/mL of sterile saline. Always prepare fresh solutions on the day of injection and do not save remaining volumes.

4. Fast mice for 6 h and inject them with LPS (injections and fasting are concomitant).
  a. At 8:00 AM, place all mice in clean cages, with fresh bedding and enrichment, and bring cages from housing to procedure room. Make sure lights are on in this new room to respect light/dark cycle, and that temperature matches that of the housing room. The procedure room must be a quiet place without circulation to people.
    ***Note:*** Removing chow from the feeder – alone – is not sufficient to grant appropriate fasting conditions. When fasting mice, transfer them to clean cages with fresh bedding and without access to food. Avoid moving cages to a different room close to starting endpoint assessment. This can stress the animals and elevate fasting blood glucose.
  b. Weigh all the mice and record body weights.
  c. Inject half of the mice pre-treated with NOD1 agonist PGN and half of the mice pre-treated with saline with 10 μL/g of body weight using LPS solution at 0.04 mg/mL (to yield a final dose of 0.4 mg/kg). If using a NOD2 agonist, inject mice with 5 μL/g of body weight using LPS solution at 0.04 mg/mL (to yield a final dose of 0.2 mg/kg). Inject the other half of the cohort with sterile saline.
  d. Wait 6 h and then proceed to endpoint assessment starting at 2:00 PM.

### Endpoint assessment – glucose tolerance test

**Timing: 2.5–3 h**

The impact of the synergy LPS-PGN on glucose tolerance is assessed by means of a GTT.

5. Measure fasting blood glucose.
  a. Use a razor blade to nick the mouse’s tail (approximately 1 cm from the tip).
  b. Gently milk the tail from bottom to top until a big drop is formed.
  c. Wipe out the first drop with gauze.
  d. Repeat the procedure and then acquire blood glucose using a glucometer and glucose strips.
  e. Record values.
6. Administer glucose load.

Inject mice intraperitoneally with 10 μL/g of body weight using a D-glucose solution at 0.2 g/mL of sterile saline (to yield a final dose of 2 g/kg).

***Note:*** For oral GTT, gavage mice with 10 μL/g of body weight using a D-glucose solution at 0.4 g/mL of sterile saline (to yield a final dose of 4 g/kg). Warm solution to 40°C and place it under constant agitation to ease glucose solubilization.

7. Monitor blood glucose after glucose load.

Apply the same procedure described in step 5 to assess blood glucose 20, 30, 40, 60, 90 and 120 min after glucose load. Record values.

## Expected outcomes

**Figure 1:**
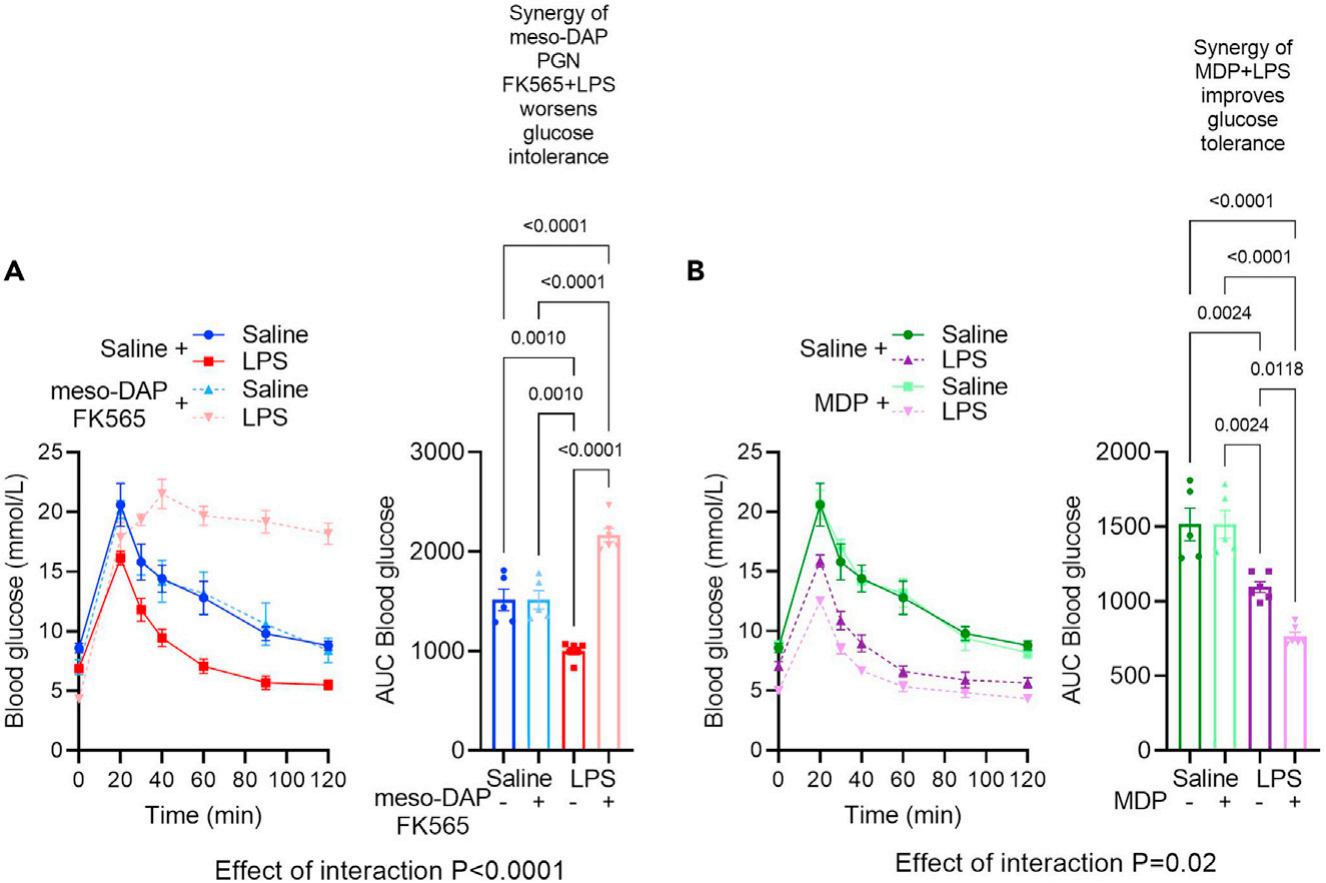
Postbiotics alter blood glucose control Exemplar data depicts the expected effect of injection of different types of peptidoglycan (PGN) and lipopolysaccharide (LPS) on blood glucose during a glucose tolerance test in mice. Exemplar data for blood glucose at each time point and area under the curve (AUC) for blood glucose and time following treatment with (A) meso-DAP containing PGN (i.e., FK565) or (B) Muramyl Dipeptide (MDP) each in combination with LPS derived from *E. coli* during an intraperitoneal (i.p.) glucose tolerance test (2 g/kg D-glucose). Note that NOD1 agonist (i.e., FK565) works in synergy with *E. coli* LPS and is expected to cause profound glucose intolerance but the NOD2 agonist (i.e., MDP) promotes tolerance and is expected to improve glucose clearance in *E. coli* LPS injected mice. Data are shown as the mean ± SEM, and the distribution of AUC values was tested using the Shapiro-Wilk test, where normality was confirmed. P values were calculated using two-way analysis of variance (ANOVA) and Tukey multiple comparison test of AUC values. Differences were considered statistically significant at P < 0.05.

PGNs are expected to synergize with LPS to alter the levels of blood glucose during a GTT. NOD1 and NOD2 agonist PGNs have opposing effects on blood glucose during LPS synergy. NOD1 agonism exacerbates (figure 4c, d in Anhê et al., 2021) and NOD2 mitigates (figure 4c in Cavallari et al., 2017) *E. coli* LPS-induced glucose intolerance (see Figure 1 for example).

## Quantification and statistical analysis

Glucose excursion curves should be plotted in XY graphs, where Y is blood glucose (mmol/L) and X is time (minutes). Calculate the area under the curves (AUCs) setting 0 as the uniform baseline for all curves. AUC is used as a readout of overall change in glucose clearance from circulation to the tissues following a glucose load. For a more in-depth understanding of AUC calculation refer to https://www.graphpad.com/guides/prism/latest/statistics/stat_area_under_the_curve.htm. Plot AUCs in a column graph (See Figure 1 for example). Test data distribution (i.e., data normality) using Shapiro-Wilk or Kolmogorov-Smirnov tests (where these options are better for small sample sizes) or Anderson-Darling or D’Agostino & Pearson tests (better for large sample sizes). These four methods are available in GraphPad Prism 9. After determining if data set is parametric or nonparametric, apply the most appropriate statistical method to compare groups. If comparing two variables at the same time (i.e., PGN and LPS), apply two-way ANOVA with Tukey’s post-test to calculate *p* values, which should be considered statistically significant at *p* < 0.05 (see Figure 4c, d in Anhê et al., 2021). Contrary to one-way ANOVA, GraphPad Prism v.9 does not offer nonparametric options with two-way ANOVA. A possible solution is to use other statistical software that performs two-way ANOVA on ranks (Kruskal Wallis test). Alternatively, it is possible to transform the values to logarithmic data, retest for normality and conduct a parametric two-way ANOVA using GraphPad Prism. If comparing only one variable at once (i.e., PGN *versus* saline where all mice received LPS injection) for AUCs, use a student t-test (parametric) or Mann-Whitney (nonparametric) test depending on data distribution (see figure 4c in Cavallari et al., 2017). We prefer to analyze AUC values to capture the cumulative effects during the entire GTT (or another dynamic test). However, a two-way repeated measures ANOVA can be used to test if blood glucose is different at specific time points between groups. The same principles (as above) should be applied to these tests, but caution is warranted in describing the relevance of a difference in blood glucose at a single time point. In some circumstances, it may be relevant to describe a difference in blood glucose at early (or late) time points after glucose or insulin injection or in other dynamic tests. However, profound changes in glucose control should be reflected in changes in the AUC. Our exemplar data (Figure 1) shows expected differences in blood glucose AUC (not just a single time point) using multiple postbiotics.

## Limitations

This method is based on acute injection(s) with postbiotics, which does not necessarily replicate chronic changes in serum concentrations found during obesity or other chronic conditions, including metabolic endotoxemia (Cani et al., 2007). Chronic infusion with osmotic minipumps is an alternative to deliver postbiotics in order to test the effects of chronically elevating the levels of specific postbiotics (see figure 7l – aa in Anhê et al., 2021). However, the latter is substantially more expensive and time-consuming.

The method described herein does not include collection of blood volume sufficient to enable the measurement of some hormones or cytokines. It is possible for experienced testers to obtain reliable blood glucose measurements and simultaneously collect blood/serum for measurement of hormones such as insulin at limited time points according to applicable ethical guidelines. Where larger blood volumes are required, reliable blood glucose measurements may not be possible, and separate cohorts of mice at each time point of testing may be required.

FK565 is a potent NOD1 agonist used to benchmark the impact of the synergy between NOD1 activators and LPS on glucose tolerance. However, FK565 may not be available commercially. Since the structure of the molecule is known, synthesizing FK565 is a possible solution. Importantly, the NOD2 agonist MDP can be easily obtained commercially and can be used to benchmark immune tolerance and improved blood glucose when used in combination with LPS from *Escherichia coli*.

## Troubleshooting

### Problem 1

Step 4: Mice may reach ethical endpoints during the experiment.

### Potential solution

Although rare, if this happens, a reduction in LPS dose should be considered.

### Problem 2

Step 4: Mice from a few cages start to fight during fasting and present higher basal blood glucose at the test.

### Potential solution

Observe mice from time to time during fasting. If you notice some of them are fighting, identify the aggressor and put it in a separate cage.

### Problem 3

Step 7: Postbiotic does not alter blood glucose control.

### Potential solution

Use new aliquots or freshly resuspended postbiotics (if obtained in powder). Test different dosages and frequency of administration. Bear in mind that some postbiotics may not directly affect blood glucose.

### Problem 4

Steps 4, 7: Injection of *E. coli* LPS lowered blood glucose during the GTT and now the scope for a postbiotic interaction that further lowers blood glucose is limited.

### Potential solution

Use new aliquots or freshly resuspended LPS. Make sure to vigorously vortex the aliquot for 15 min prior to use. Consider using lower doses of LPS.

### Problem 5

Step 7: When analyzing blood glucose curves, some mice seem to respond differently to LPS stimulation.

### Potential solution

Make sure there is no cage effect related, for example, to fighting during fasting or other uncontrolled variable. Make sure LPS is correctly delivered intraperitoneally. Common mistakes during this technique include injection into the bladder or liquid reflux after injection.

## Resource availability

### Lead contact

Further information and requests for resources and reagents should be directed to and will be fulfilled by the lead contact, Jonathan D. Schertzer (schertze@mcmaster.ca).

### Materials availability

This work did not generate new unique reagents and all resources used in the current study are commercially available.

## Data Availability

The datasets generated and/or analyzed during the current study are available from the corresponding author on reasonable request. This study did not generate/analyze codes.
